# 基于人工智能影像学特征参数构建部分实性肺结节良恶性预测模型的应用价值

**DOI:** 10.3779/j.issn.1009-3419.2025.102.13

**Published:** 2025-04-20

**Authors:** Mingzhi LIN, Yiming HUI, Bin LI, Peilin ZHAO, Zhizhong ZHENG, Zhuowen YANG, Zhipeng SU, Yuqi MENG, Tieniu SONG

**Affiliations:** 730030 兰州，兰州大学第二医院（第二临床医学院）胸外科; Department of Thoracic Surgery, The Second Hospital & Clinical Medical School, Lanzhou University, Lanzhou 730030, China

**Keywords:** 肺肿瘤, 部分实性肺结节, 人工智能, 机器学习, 预测模型, Lung neoplasms, Part-solid nodule, Artificial intelligence, Machine learning, Prediction model

## Abstract

**背景与目的:**

肺癌是全球最常见的恶性肿瘤之一，也是癌症相关死亡的主要原因。早期肺癌通常表现为肺结节，准确评估其恶性风险对于延长生存期及避免过度诊疗至关重要。本研究旨在基于人工智能（artificial intelligence, AI）自动提取的影像学特征参数构建模型，评估其在部分实性结节（part-solid nodule, PSN）恶性预测中的效能。

**方法:**

回顾性分析2020年10月至2025年2月于兰州大学第二医院接受肺结节切除术的222例患者的229个PSN资料。根据病理结果，将45个良性病变及腺体前驱病变归为非恶性组，184个肺部恶性肿瘤归为恶性组。所有患者均接受胸部计算机断层扫描，使用AI软件提取影像学特征参数。通过单因素分析筛选显著变量，计算方差膨胀因子并剔除共线性较高的变量，LASSO回归进一步筛选关键特征，多因素逻辑回归确定独立危险因素。基于筛选结果，构建逻辑回归、随机森林、XGBoost、LightGBM、支持向量机5种模型，使用受试者工作特征（reciever operating characteristic, ROC）曲线评估模型性能。

**结果:**

PSN良恶性的独立危险因素包括粗糙度（ngtdm）、依赖方差（gldm）和短运行低灰度重点（glrlm）。逻辑回归在训练集和测试集的曲线下面积（area under the curve, AUC）分别为0.86和0.89，表现较好。XGBoost的AUC分别为0.78和0.77，表现相对均衡，但准确度较低。支持向量机在训练集的AUC为0.93，测试集AUC降至0.80，表明该模型存在一定的过拟合。LightGBM在训练集表现优异，AUC为0.94，但在测试集上有所下降，AUC为0.88。随机森林模型在训练集和测试集上均表现稳定，训练集AUC为0.89，测试集AUC为0.91，具有较高的稳定性和良好的泛化能力。

**结论:**

基于独立危险因素构建的随机森林模型在PSN良恶性预测中表现最佳，可以为临床医生提供有效的辅助预测，支持个体化治疗决策。

## Abstract (en)

**Background and objective:**

Lung cancer is one of the most common malignant tumors worldwide and a major cause of cancer-related deaths. Early-stage lung cancer is often manifested as pulmonary nodules, and accurate assessment of the malignancy risk is crucial for prolonging survival and avoiding overtreatment. This study aims to construct a model based on image feature parameters automatically extracted by artificial intelligence (AI) to evaluate its effectiveness in predicting the malignancy of part-solid nodule (PSN).

**Methods:**

This retrospective study analyzed 229 PSN from 222 patients who underwent pulmonary nodule resection at Lanzhou University Second Hospital between October 2020 and February 2025. According to pathological results, 45 cases of benign lesions and precursor glandular lesion were categorized into the non-malignant group, and 184 cases of pulmonary malignancies were categorized into the malignant group. All patients underwent preoperative chest computed tomography (CT), and AI software was used to extract imaging feature parameters. Univariate analysis was used to screen significant variables; variance inflation factor (VIF) was calculated to exclude highly collinear variables, and LASSO regression was further applied to identify key features. Multivariate Logistic regression was used to determine independent risk factors. Based on the selected variables, five models were constructed: Logistic regression, random forest, XGBoost, LightGBM, and support vector machine (SVM). Receiver operating characteristic (ROC) curves were used to assess the performance of the models.

**Results:**

The independent risk factors for the malignancy of PSN include roughness (ngtdm), dependence variance (gldm), and short run low gray-level emphasis (glrlm). Logistic regression achieved area under the curves ( AUCs) of 0.86 and 0.89 in the training and testing sets, respectively, showing good performance. XGBoost had AUCs of 0.78 and 0.77, respectively, demonstrating relatively balanced performance, but with lower accuracy. SVM showed an AUC of 0.93 in the training set, which decreased to 0.80 in the testing set, indicating overfitting. LightGBM performed excellently in the training set with an AUC of 0.94, but its performance declined in the testing set, with an AUC of 0.88. In contrast, random forest demonstrated stable performance in both the training and testing sets, with AUCs of 0.89 and 0.91, respectively, exhibiting high stability and excellent generalizability.

**Conclusion:**

The random forest model constructed based on independent risk factors demonstrated the best performance in predicting the malignancy of PSN and could provide effective auxiliary predictions for clinicians, supporting individualized treatment decisions.

肺癌是最常见的恶性肿瘤之一，也是全球致死率最高的癌症，据统计2022年全球肺癌的总死亡人数为181.7万，位居所有癌症死亡人数之首^[1]^。早期肺癌常表现为肺结节，按照密度分为磨玻璃结节（ground glass nodule, GGN）和实性结节（solid nodule, SN）。其中，GGN根据是否含有实性成分分为纯磨玻璃结节（pure GGN, pGGN）和部分实性结节（part-solid nodule, PSN）^[2]^。GGN的术前诊断方法包括侵入性和非侵入性检测。根据美国国立综合癌症网络（National Comprehensive Cancer Network, NCCN）指南，低剂量胸部计算机断层扫描（low-dose computed tompgraphy, LDCT）被推荐为肺结节筛查的首选方法，能够有效促进肺癌的早期发现、诊断和治疗，进而降低肺癌的死亡率^[3]^。与SN相比，LDCT、正电子发射计算机断层显像（positron emission tomography, PET）和非手术活检对PSN的诊断相对不敏感^[4]^。目前，PSN的恶性程度评估主要依赖经验丰富的影像学医师及胸外科医师，但受主观判断、影像分辨率和图像噪声等因素影响，其准确性较为有限。此外，LDCT的重复检查缺乏一致性，难以确保数据可比性。由于高昂费用和有限的评估价值，PET/CT适用范围相对局限。侵入性检测方法（如肺穿刺活检和支气管镜取样）虽可用于病理确诊，但对<1 cm的结节和深部病变，操作难度大，且易引发气胸、肺内出血等并发症，存在较高的假阴性风险^[5]^。

此前研究^[6]^发现，PSN相较于其他类型的GGN显示出更高的恶性程度，且其总生存率较低，5年复发率较高。尽管PSN的恶性概率较高，但仍有部分PSN表现为良性病变。因此在临床实践中，术前准确评估PSN的良恶性至关重要。随着图像技术的进步和医学领域的发展，基于深度学习和大数据构建的人工智能（artificial intelligence, AI）辅助诊断软件在多个医学领域得到了广泛应用。AI能够自动提取和分析CT图像中的影像学特征，智能识别并预测肺结节的良恶性。其较高的敏感性和可重复性有助于辅助临床医生进行早期诊断，减少误诊与漏诊^[7]^，从而提高患者的生存质量并改善预后。本研究旨在探讨AI自动化提取的影像学特征参数预测PSN良恶性的可行性和临床应用价值。

## 1 资料与方法

### 1.1 临床资料和分组

纳入标准：（1）具有完整的肺结节切除手术记录和术后完整病理报告；（2）术前1周内行CT检查且图像层厚≤1.25 mm且诊断为PSN；（3）术前未行抗肿瘤治疗。排除标准：（1）图像质量欠佳或有严重伪影；（2）术前有恶性肿瘤病史或已行其他对症治疗；（3）结节被证实为其他部位肿瘤转移灶。

纳入2020年10月至2025年2月兰州大学第二医院收治的222例患者，共229个PSN。将良性病变+腺体前驱病变[其余良性病变25个]归为非恶性组，肺部恶肿瘤（184个，其中浸润性非黏液腺癌179个，细支气管肺泡癌1个，浸润性黏液腺癌4个）归为恶性组。该回顾性研究遵循相关法律并获得伦理批准（批准号：2024A-919）。所有数据均匿名处理，去标识化后不包含患者的个人信息，避免反向识别；且数据访问严格限制，仅授权研究人员可获取。

### 1.2 检查方法

CT影像使用了以下几种系统：Discovery CT750 HD（GE Healthcare）、Philips iCT 256（Koninklijke Philips N.V.）和Somatom Sensation 64（西门子，德国埃尔朗根）。扫描设置如下：管电压设定为120 kVp，管电流为150-200 mA。轴向图像的层厚和层间距均保持为5 mm，重建层厚和间隔均设定为1.25 mm。所有CT图像的分析采用了肺窗设置（窗宽：1500 HU；窗位：-600 HU）。患者采集胸部薄层CT图像后将DICOM文件导入AI系统（深睿Dr. Wise®肺结节AI医学辅助诊断系统，深睿博联科技有限公司，北京），由AI系统自动定位结节位置，勾画感兴趣区域（region of interest, ROI）并提取自动化参数。所有处理过程均为自动化完成，勾画靶区不涉及人工干预（图1）。

**图1:**
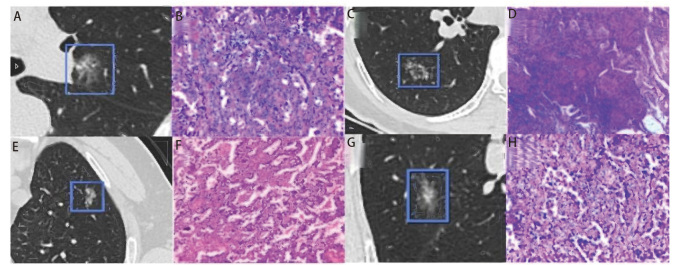
人工智能系统自动识别的PSN与相应病理。非恶性组A、B：硬化细胞瘤（图1B，HE，×200）；C、D：结核瘤（图1D，HE，×200）。恶性组E、F：中分化腺癌（图1F，HE，×200）；G、H：微小浸润腺癌（图1H，HE，×200）。

### 1.3 提取参数

由AI系统自动批量提取总计122个影像学特征参数，其中114个影像组学特征，包括形状特征，如结节长径、结节短径、总质量、总体积等；灰度特征，如平均CT值、最大CT值、最小CT值熵、强度（ngtdm）等；纹理特征包括繁忙度（ngtdm）、粗糙度（ngtdm）、依赖方差（gldm）等。8个结节影像学语义特征，如支气管截断征、不规则、伴钙化、血管包被、胸膜牵拉等。

### 1.4 统计分析

对于连续变量，根据其是否符合正态分布分别采用t检验和Wilcoxon秩和检验评估不同组间的差异。对于分类变量，通过卡方检验或Fisher精确检验评估其在不同组间的分布差异，对正态分布的计量资料采用均数±标准差（Mean±SD）描述，不符合正态分布的计量资料采用四分位数（Q1, Q3）描述。计算变量间的方差膨胀因子（variance inflation factor, VIF）评估共线性，剔除VIF值>5的变量。此外，采用单因素回归分析评估每个独立变量对因变量的影响。使用LASSO回归分析，结合五折交叉验证和网格搜索进行超参数调优和特征选择。对剩余变量构建多因素回归模型，确定独立危险因素。采用逻辑回归、随机森林、XGBoost、LightGBM、支持向量机（support vector machine, SVM）5种算法构建最终模型。对数据按照0.75和0.25划分训练集和测试集，对训练集使用十折交叉验证与网格搜索来确定最佳超参数，并根据受试者工作特征（reciever operating characteristic, ROC）曲线灵敏度、特异度评价模型的区分度和临床效用。所有统计分析均在R 4.4.2、IBM SPSS Statistics 26和Empower Stats软件中完成。P<0.05为差异有统计学意义。

## 2 结果

### 2.1 两组结节参数的组间比较

本研究共纳入229个PSN，其中非恶性45个，恶性184个，其中非恶性组平均年龄52.96岁，恶性组为58.24岁，年龄是PSN良恶性的显著影响因素（P<0.01），分析结果显示不规则、支气管截断征、胸膜牵拉、病灶位置、总质量、总体积、长径、短径、相关性（glcm）、最小轴长度（shape）及最大相关系数（glcm）等69个影像学特征参数差异均有统计学意义（P均<0.05，详见表1、表2）。性别（P=0.36）和实性成分占比（consolidation tumor ratio, CTR）（P=0.58）在良恶性结局中无统计学差异。

**表1:** 两组患者一般临床资料比较

Variable	Non-malignant group (n=45)	Malignant group (n=184)	P
Age (Mean±SD) (yr)	52.96±10.63	58.24±8.84	<0.01
Gender			0.36
Female	27 (60.0%)	126 (68.5%)	
Male	18 (40.0%)	58 (31.5%)	
Lesion location			<0.01
Right upper lobe	10 (22.2%)	68 (37.0%)	
Right middle lobe	4 (8.9%)	15 (8.2%)	
Right lower lobe	19 (42.2%)	29 (15.8%)	
Left upper lobe	8 (17.8%)	53 (28.8%)	
Left lower lobe	4 (8.9%)	19 (10.3%)	
Bronchial truncation sign			<0.01
No	43 (95.6%)	112 (60.9%)	
Yes	2 (4.4%)	72 (39.1%)	
Irregular			0.01
No	37 (82.2%)	113 (61.4%)	
Yes	8 (17.8%)	71 (38.6%)	
Pleural retraction			0.03
No	44 (97.8%)	160 (87.0%)	
Yes	1 (2.2%)	24 (13.0%)	
CTR	0.50 (0.37, 0.69)	0.53 (0.40, 0.66)	0.58

CTR: consolidation tumor ratio.

**表2:** 两组患者影像学特征参数

Variable	Non-malignant group (n=45)	Malignant group (n=184)	P
Long axis	10.00 (8.00, 16.50)	18.50 (14.00, 24.00)	<0.01
Short axis	7.50 (6.00, 11.75)	14.00 (10.00, 18.00)	<0.01
Run entropy (glrlm)	2.90±0.40	3.20±0.30	<0.01
Large dependence high (gldm)	298.50±63.60	354.30±62.20	<0.01
Total volume	442.41 (331.19, 1084.21)	2057.18 (954.98, 3534.99)	<0.01
Total mass	243.35 (144.62, 600.25)	944.30 (448.80, 2022.02)	<0.01
CT max value	240.00 (58.75, 320.75)	340.50 (236.25, 445.25)	<0.01
CT min value	-1009.50 (-1024.00, -923.75)	-1024.00 (-1024.00, -1024.00)	<0.01
Maximum 2D area	54.06 (36.19, 143.02)	182.57 (107.76, 284.75)	<0.01
Surface area	281.14 (204.24, 568.10)	744.38 (483.52, 1253.30)	<0.01
Energy	4.70×10^10^ (2.50×10^10^, 5.65×10^11^)	1.20×10^12^ (3.58×10^11^, 5.50×10^12^)	<0.01
CT value variance	51,882.80 (44,578.28, 77,737.33)	68,420.45 (53,669.15, 87,611.08)	<0.01
3D long axis	13.22 (10.58, 19.21)	21.23 (16.41, 27.71)	<0.01
Compactness	0.51 (0.42, 0.66)	0.64 (0.54, 0.78)	<0.01
Average of long and short axes	9.00 (7.00, 14.50)	17.00 (13.00, 21.00)	<0.01
Entropy	8.46 (7.95, 9.11)	9.44 (9.04, 9.75)	<0.01
Busyness (ngtdm)	22.76 (13.78, 123.68)	143.76 (74.61, 330.72)	<0.01
Large area low gray level emphasis (glrlm)	6687.89 (2887.13, 159,500.98)	220,795.34 (63,866.97, 716,401.87)	<0.01
Short-axis length (shape)	8.98 (7.86, 12.14)	14.91 (10.94, 18.58)	<0.01
Large area high gray level emphasis (glrlm)	9.72×10^4 ^(4.23×10^4^, 9.95×10^5^)	1.96×10^6^ (5.38×10^5^, 6.21×10^6^)	<0.01
Roughness (ngtdm)	0.02 (0.00, 0.03)	0.00 (0.00, 0.01)	<0.01
Gray level non-uniformity 1 (glrlm)	121.18 (80.81, 335.73)	449.06 (259.56, 899.15)	<0.01
Large dependence high gray level emphasis (gldm)	1064.29 (854.51, 1243.10)	1316.97 (1125.39, 1478.45)	<0.01
Long run high gray level emphasis (glrlm)	32.00 (25.29, 46.87)	50.60 (39.79, 69.02)	<0.01
Maximum 2D diameter (Column) (shape)	13.86 (10.96, 19.40)	19.48 (15.83, 25.84)	<0.01
Minor axis length (shape)	6.99 (5.59, 8.26)	11.16 (9.12, 14.37)	<0.01
Strength (ngtdm)	0.02 (0.00, 0.03)	0.00 (0.00, 0.01)	<0.01
Maximum 2D diameter (Row) (shape)	13.43 (10.59, 18.43)	19.59 (15.87, 25.68)	<0.01
Small dependence high gray level emphasis (gldm)	0.03 (0.02, 0.04)	0.02 (0.02, 0.02)	<0.01
Surface area to volume ratio (shape)	0.83 (0.70, 0.94)	0.52 (0.43, 0.66)	<0.01
Long axis length (shape)	12.58 (10.10, 17.80)	18.49 (14.71, 24.21)	<0.01
Long run low gray level emphasis (glrlm)	3.42 (2.62, 4.49)	5.15 (4.04, 6.79)	<0.01
Voxel volume (shape)	442.92 (331.19, 1083.46)	2057.33 (955.59, 3533.20)	<0.01
Maximum 2D diameter-slice (shape)	11.69 (9.81, 18.19)	20.20 (15.19, 26.21)	<0.01
Large area emphasis (glrlm)	24,813.78 (10,727.11, 326,569.25)	572,389.70 (152,641.21, 1,808,619.19)	<0.01
Short run high gray level emphasis (glrlm)	1.10 (1.01, 1.22)	0.97 (0.89, 1.06)	<0.01
Gray level non-uniformity normalized 1 (glrlm)	0.51 (0.50, 0.53)	0.51 (0.50, 0.51)	<0.01
Small dependence emphasis (gldm)	0.02 (0.01, 0.03)	0.01 (0.01, 0.01)	<0.01
Grid volume (shape)	432.02 (322.61, 1064.50)	2042.05 (941.06, 3507.52)	<0.01
High gray level run emphasis (glrlm)	2.63 (2.44, 2.86)	2.41 (2.34, 2.54)	<0.01
Low gray level run emphasis (glrlm)	0.59 (0.53, 0.64)	0.65 (0.62, 0.67)	<0.01
Total energy (firstorder)	756.57 (521.06, 1423.30)	2540.36 (1203.25, 5404.73)	<0.01
Dependence non-uniformity (gldm)	26.96 (15.20, 79.27)	134.89 (63.66, 325.24)	<0.01
Gray level non-uniformity (gldm)	372.38 (214.59, 863.23)	1357.42 (752.07, 2951.51)	<0.01
Large dependence low gray level emphasis (gldm)	94.17 (77.42, 119.92)	114.23 (101.00, 131.41)	<0.01
Run length non-uniformity (glrlm)	61.73 (47.08, 198.26)	240.12 (149.93, 464.43)	<0.01
Area percentage (glrlm)	0.01 (0.00, 0.03)	0.00 (0.00, 0.01)	<0.01
Run percentage (glrlm)	0.44 (0.36, 0.49)	0.37 (0.33, 0.40)	<0.01
Area variance (glrlm)	19,969.55 (8348.81, 65,333.72)	328,919.88 (76,313.77, 1,262,906.12)	<0.01
Variable	Non-malignant group (n=45)	Malignant group (n=184)	P
Gray level variance 2 (glrlm)	0.24 (0.23, 0.25)	0.25 (0.24, 0.25)	<0.01
Long run emphasis (glrlm)	9.25 (7.21, 13.03)	14.21 (11.60, 19.50)	<0.01
Run variance (glrlm)	3.17 (2.33, 4.77)	6.29 (4.40, 9.42)	<0.01
Inverse maximum correlation (glcm)	-0.06 (-0.12, -0.03)	-0.08 (-0.12, -0.05)	0.04
Run length non-uniformity normalized (glrlm)	0.29±0.06	0.27±0.04	0.04
Small area low gray level emphasis (glrlm)	0.31 (0.16, 0.41)	0.37 (0.23, 0.49)	0.04
Inverse maximum correlation 2 (glcm)	0.26 (0.17, 0.43)	0.32 (0.26, 0.40)	0.04
Maximum correlation coefficient (glcm)	0.23 (0.16, 0.38)	0.29 (0.24, 0.37)	0.03
Correlation (glcm)	0.23 (0.12, 0.38)	0.29 (0.24, 0.37)	0.01
Small dependence low gray level emphasis (gldm)	0.01 (0.01, 0.02)	0.01 (0.01, 0.01)	<0.01
Dependence variance (gldm)	46.48 (40.05, 52.16)	49.65 (43.97, 56.11)	0.01
Short run low gray level emphasis (glrlm)	0.38 (0.34, 0.42)	0.41 (0.38, 0.43)	0.01
Dependence non-uniformity normalized (gldm)	0.05 (0.05, 0.05)	0.05 (0.05, 0.07)	<0.01
Sphericity	0.81±0.11	0.86±0.08	<0.01
Cluster shade (glcm)	-0.26 (-0.32, -0.20)	-0.32 (-0.36, -0.27)	<0.01

Due to the large number of variables, only the significant variables with P<0.05 are retained Tab 2. The letters in parentheses in Tab 2 represent feature categories, not abbreviations. CT: computed tompgraphy.

### 2.2 VIF与LASSO回归优化变量选择

为了验证变量之间是否存在多重共线性问题，我们计算了VIF，剔除了VIF>5的变量，保留19个变量。为了进一步筛选合适的变量，我们进行了单因素回归分析，保留具有统计学意义的16个变量（表3）。随后，使用LASSO回归进一步筛选变量。最终得到10个变量：短径、年龄、CT最小值、粗糙度（ngtdm）、依赖方差（gldm）、短运行高灰度重点（glrlm）、小面积低灰度重点（glrlm）、聚类阴影（glcm）、依赖非一致性归一化（gldm）、短运行低灰度重点（glrlm）。

**表3:** 单因素逻辑回归和多因素逻辑回归

Variable	Univariate Logistic regression					Multivariable Logistic regression			
	β	OR (95%CI)	Z	P		β	OR (95%CI)	Z	P
Age	0.06	1.06 (1.02-1.10)	3.26	<0.01		0.03	1.03 (0.99-1.08)	1.33	0.18
Short axis	0.20	1.23 (1.13-1.33)	4.75	<0.01		0.05	1.05 (0.93-1.19)	0.80	0.42
CT min value	-0.02	0.99 (0.98-0.99)	-4.39	<0.01		-0.01	0.99 (0.98-1.00)	-1.40	0.16
Roughness (ngtdm)	-84.60	0.00 (0.00-0.00)	-5.38	<0.01		-53.56	0.00 (0.00-0.63)	-1.98	0.04
Dependence variance (gldm)	0.05	1.05 (1.01-1.09)	2.71	<0.01		0.08	1.09 (1.02-1.16)	2.63	<0.01
Dependence non-uniformity normalized (gldm)*	0.42	1.53 (1.14-2.06)	1.72	<0.01		0.42	1.52 (0.94-2.45)	1.72	0.08
Short run high gray level emphasis (glrlm)	-5.67	0.00 (0.00-0.04)	-4.73	<0.01		-1.16	0.31 (0.01-10.92)	-0.64	0.52
Small area low gray level emphasis (glrlm)*	1.96	7.08 (1.19-42.00)	2.15	<0.01		0.03	1.03 (1.00-1.06)	1.88	0.06
Short run low gray level emphasis (glrlm)*	0.11	1.12 (1.04-1.20)	3.05	<0.01		0.14	1.15 (1.02-1.29)	2.35	0.01
Cluster shade (glcm)*	-3.83	0.02 (0.00-0.39)	-2.60	<0.01		0.03	1.03 (0.99-1.08)	1.50	0.13
Bronchial truncation sign	0.20	1.22 (0.60-2.49)	0.55	0.58					
Irregular	-0.30	0.74 (0.38-1.45)	-0.86	0.38					
Pleural retraction	0.64	1.90 (0.54-6.66)	1.00	0.31					
Correlation (glcm)	3.09	21.87 (1.11-430.76)	2.03	0.04					
CT max value	0.01	1.01 (1.01-1.01)	2.36	0.01					
CT value variance	0.01	1.01 (1.01-1.01)	2.94	<0.01					
Gray level non-uniformity normalized 1 (glrlm)	-28.70	0.00 (0.00-0.00)	-3.72	<0.01					
Dependence non-uniformity (gldm)	0.01	1.01 (1.01-1.01)	2.52	0.01					
Small dependence low gray level emphasis (gldm)	-82.62	0.00 (0.00-0.00)	-3.56	<0.01					

*indicates that the variable has been magnified by a factor of 100. OR: odds ratio.

### 2.3 逐步筛选独立危险因素

对筛选出的变量进行多因素回归分析（表3），结果显示粗糙度（ngtdm）、依赖方差（gldm）和短运行低灰度重点（glrlm）具有统计学差异（P<0.05），而其他因素（如年龄、短径、CT最小值等）未显示出明显差异。根据单因素及多因素回归分析最终确定了粗糙度（ngtdm）、依赖方差（gldm）、短运行低灰度重点（glrlm）作为区分PSN良恶性的独立危险因素。

### 2.4 构建PSN良恶性预测模型

应用5种机器学习模型（逻辑回归、LightGBM、随机森林、SVM、XGBoost）评估模型的性能（表4）并绘制了ROC曲线（图2）和校准曲线（图3）。其中LightGBM在训练集上表现最佳，曲线下面积（area under the curve, AUC）为0.94，显示出较高的预测能力。随机森林在测试集上表现最佳，AUC为0.91，展现了良好的泛化能力。同时随机森林在训练集和测试集上的表现都较为均衡，且灵敏度、特异度、准确度和精确度之间的平衡最佳。其余结果详见表4。在校准曲线中，LightGBM、逻辑回归和随机森林模型表现较好，其预测与实际结果的吻合度较高。SVM和XGBoost的校准效果稍差，显示出一定的预测偏差，因此，随机森林在所有模型中表现最为可靠。

**表4:** 5种模型的预测效能比较

Model		AUC	95%CI	P	Cut-off value	Sensitivity	Specificity	Accuracy	Precision
Logistic regression	Training set	0.86	0.79-0.92	0.62	0.78	0.76	0.85	0.78	0.95
	Testing set	0.89	0.78-1.00		0.78	0.91	0.75	0.88	0.93
XGBoost	Training set	0.78	0.71-0.86	0.84	0.79	0.78	0.67	0.75	0.91
	Testing set	0.77	0.65-0.90		0.79	0.78	0.67	0.79	0.91
SVM	Training set	0.93	0.90-0.96	0.11	0.80	0.86	0.91	0.82	0.91
	Testing set	0.80	0.67-0.95		0.80	0.80	0.67	0.83	0.90
LightGBM	Training set	0.94	0.91-0.97	0.23	0.82	0.79	0.99	0.83	0.99
	Testing set	0.88	0.77-0.98		0.82	0.76	0.75	0.76	0.92
Random forest	Training set	0.89	0.83-0.94	0.67	0.88	0.82	0.85	0.82	0.96
	Testing set	0.91	0.82-0.99		0.88	0.85	0.75	0.83	0.93

The model uses a 0.75 and 0.25 split for the training set and testing set. The training set undergoes 10-fold cross-validation, and grid search is used to determine the best parameters. AUC: area under the curve.

**图2:**
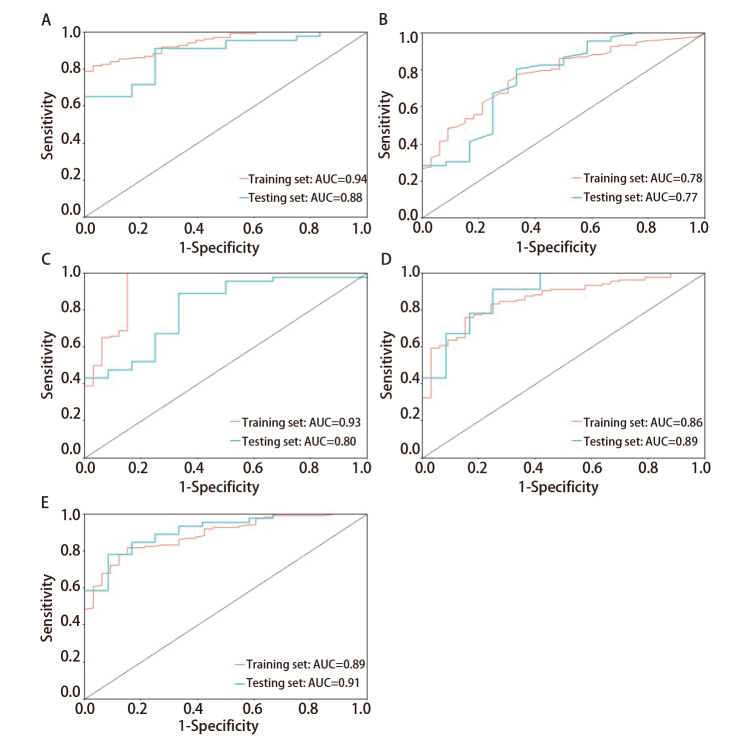
5种机器学习模型的ROC曲线。A：LightGBM；B：XGBoost；C：支持向量机；D：逻辑回归；E：随机森林。

**图3:**
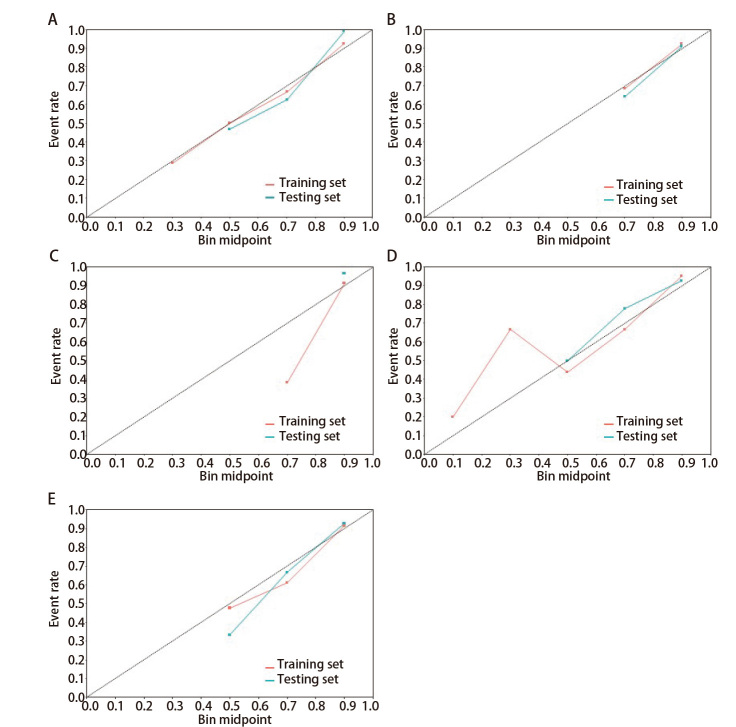
5种机器学习模型的校准曲线。A：LightGBM；B：XGBoost；C：支持向量机；D：逻辑回归；E：随机森林。

## 3 讨论

作为全球发病率最高的癌症之一，肺癌的早期发现和诊断对提高患者生存率至关重要^[1]^。随着深度学习和计算机辅助诊断技术的发展，基于CT图像的AI辅助诊断系统为肺结节的良恶性预测提供了有效的辅助诊断手段^[7]^。本研究旨在构建一个基于AI自动提取影像学特征参数的PSN良恶性预测模型。在研究过程中，我们共纳入了122个影像学特征参数，结果显示，粗糙度（ngtdm）、依赖方差（gldm）和短运行低灰度重点（glrlm）是预测PSN良恶性的独立危险因素。基于这些筛选出的影像学纹理特征，我们构建了预测模型。这些纹理特征通过量化结节的形态学和灰度变化模式，捕捉结节的“微观”结构，反映了结节内部的组织异质性和结构复杂性，已被证明与结节的恶性潜力相关^[8]^。粗糙度（ngtdm）是邻域灰度差异矩阵的特征之一，邻域灰度差异矩阵反映了结节表面纹理的复杂性，通常由结节表面像素灰度的波动性决定^[9]^。依赖方差（gldm）是灰度依赖矩阵的重要特征之一，灰度依赖矩阵衡量像素之间的空间依赖关系。恶性结节通常呈现较大的灰度变化，反映出肿瘤组织的不均匀性^[10]^。影像学上，恶性结节常表现为增强不均匀性和更显著的组织异质性，而良性结节通常较为均匀。Prasla等^[11]^研究表明，灰度依赖矩阵特征可用于评估肿瘤异质性，与肿瘤的恶性进展密切相关。短运行低灰度重点（glrlm）属于灰度级运行长度矩阵的特征。灰度级运行长度矩阵能够提供关于组织连续性和均匀性的重要信息。通过计算图像中低灰度区域的运行长度，灰度级运行长度矩阵描述像素在图像中的排列方式，这对于肿瘤组织的结构分析具有重要价值^[12]^。相关研究^[13,14]^发现灰度级运行长度矩阵在肿瘤检测与分类中具有重要作用，特别是在脑肿瘤、乳腺癌等类型的肿瘤分析中恶性肿瘤的灰度级运行长度矩阵特征表现为较低的同质性和较高的纹理不规则性，表明恶性肿瘤具有更加复杂的组织结构和不连续的纹理特征。

在胸部CT影像诊断中，放射科医师通常根据肺结节的大小，边缘特征和CTR来评估结节的良恶性^[15]^。这些影像学特征需要结合动态观察（如倍增时间）以及医师的经验进行综合判断，但是蕴含在数字化图像中的大量像素纹理特征信息未被充分利用。随着放射组学作为新兴领域的快速发展，它在从医学图像中提取高通量数据方面以及在癌症早期诊断和个体化治疗研究中的应用潜力展现巨大。近年来，部分研究^[16,17]^探索了基于影像组学特征的建模策略，通过定量分析CT影像组学特征或联合临床变量，构建非侵入性预测工具以评估肺结节的恶性风险。Warkentin等^[18]^基于四项国际肺癌筛查队列（共6865例，包含513个恶性结节）开发了一个整合642个放射组学特征和9项流行病学因素的机器学习模型。该模型在测试集上表现优异（AUC=0.93, 95%CI: 0.90-0.96），显著优于Pan-Canadian模型（AUC=0.87），并对SN（AUC=0.93）与亚实性结节（AUC=0.91）均具有较高的预测能力，表明其在肺结节恶性风险分层中的可靠性。Liu等^[19]^纳入875例肺结节患者（训练队列n=612，验证队列n=263），通过LASSO算法从1288个放射组学特征中筛选出20个关键特征，结合年龄构建了多因素逻辑回归列线图。该模型在训练集（AUC=0.83）和验证集（AUC=0.80）中均表现出良好的区分度，校准曲线与Hosmer-Lemeshow检验（P>0.05）验证了其稳定性，提示该列线图可为肺癌筛查中的肺结节良恶性鉴别提供有效支持。

本研究分析在模型构建过程中纳入的最终变量均为AI软件自动提取的影像组学参数，致力于避免观察者间误差。接着，我们使用不同的机器学习构建预测模型并选择最优模型。逻辑回归在训练集和测试集的AUC分别为0.86和0.89，提示具有较好的预测性能，灵敏度（训练集0.76，测试集0.91）和特异度（训练集0.85，测试集0.75）表现尚可。XGBoost在训练集和测试集上的AUC分别为0.78和0.77，灵敏度表现较为均衡（均为0.78），但在特异度方面较低（均为0.67）。SVM在训练集上表现较好（AUC=0.93），但测试集性能下降明显（AUC=0.80），训练集的灵敏度和特异度表现较好（0.86和0.91），但测试集表现不佳（0.80和0.67）。LightGBM模型在训练集表现最佳（AUC=0.94），但测试集性能下降（AUC=0.88）。相比之下，随机森林模型在训练集（AUC=0.89）表现较好，测试集表现最佳（AUC=0.91），灵敏度（训练集0.82，测试集0.85）和特异度（训练集0.85，测试集0.75）均较为平衡，表现出良好的泛化能力。随机森林模型在本研究中表现出较高的稳定性和最佳的预测性能，因此被评为优选模型。通过多次构建决策树并优化结果，随机森林模型能够有效处理高维数据，避免过拟合问题。同时提示使用AI软件自动提取的影像组学参数构建的预测模型，能够为PSN的良恶性预测提供稳定、可靠的依据，并展示出良好的预测能力。

在肺结节影像组学研究中，参数提取的可重复性与效率是影响模型临床适用性的关键因素。传统半自动化参数提取依赖医师手动勾画病灶边界（如ITK-SNAP或3D Slicer软件），这种方法存在两大局限性：首先是不同观察者间的勾画差异会显著影响放射组学特征的可重复性。例如，Liu等^[20]^发现，半自动勾画肺结节时，不同医师的组内相关系数（intra-class correlation coefficient, ICC）仅为0.65-0.82，尤其在边界模糊病灶中差异更大；另一方面，人工标注耗时且效率低下，限制了大样本研究的可行性^[21]^。本研究采用深睿Dr. Wise® AI系统实现全自动化定位结节位置、勾画ROI并进行参数提取，其优势体现在提升可重复性，提高效率与标准化处理。AI算法通过端到端深度学习统一分割标准，消除人为偏差。研究^[18]^表明，自动化分割的放射组学特征ICC值普遍>0.95，显著高于半自动化方法。类似地，Ahn等^[16]^使用自动勾画系统（基于深度学习算法）在肝脏和脾脏的分割及体积测量中具有显著优势。其自动化程度高，准确性强，Dice相似度系数超过0.97，体积测量与手动分割的偏差较小（肝脏体积偏差为-0.17%）。自动化处理在不需要人工修正的同时可适配多中心数据异质性（如扫描协议差异），通过标准化预处理（体素重采样、灰度归一化）提升跨机构数据的可比性^[22]^。此外，全自动化系统提取速度和精度更高，可在5 s内完成单例CT图像的结节分割及上千个参数的提取（包括形态学、纹理及高阶小波特征），而传统方法需依赖医师逐层修正^[17]^。

本研究使用一个包含经病理证实的单一PSN的数据集，显示了AI自动提取影像学特征参数作为PSN良恶性判断的无创预测因子的潜力。通过多元回归分析，解释潜在的混杂因素，并从候选变量中筛选出与恶性病变最显著相关的影像学特征参数。借助先进的AI分析软件，我们实现了对影像学特征参数的高效精确的提取和定量分析。在此基础上，采用多种机器学习算法构建了基于影像学特征参数的PSN恶性预测模型，并筛选出性能最优的模型。该模型可能为临床医生提供一个客观精确的辅助诊断工具，有助于提高PSN恶性风险预测的准确性，减少恶性风险较高的PSN的过度诊疗。

构建共享、开放、标准统一且具本土特色的多病种、多模态、多中心医学影像数据库，是AI技术发展的重要方向，也是推动医学影像AI落地和临床转化的关键保障^[23]^。AI作为新一代科技革命的重要产物，仍处于发展初期，其功能有待进一步完善和提升，未来的研究可能通过结合多模态数据来实现更准确的预测。除了传统的CT影像数据外，PET/CT能够提供结节的代谢信息，结合这两种影像学数据将有助于更准确地判断结节的恶性程度^[24]^。此外，血清标志物在肿瘤的早期筛查中具有重要价值^[25]^，将其与影像学特征相结合，可能提高模型的敏感性和特异性。我们希望未来研究者可以结合多中心的大样本量多模态参数，使用更先进的技术手段，将来自不同数据源的信息融合，构建可供不同中心统一使用的肺结节AI软件。

本研究尚存在一些局限性：（1）仅在单中心数据中进行交叉验证，其结果的普适性和可重复性尚不明确。因此，未来有必要开展多中心、前瞻性研究，以进一步验证模型的可靠性和泛化能力；（2）作为一项回顾性研究所有数据依赖于已有记录，可能存在选择和信息偏倚，从而导致数据不完整。
